# General and Specific Effects of Stereo Learning

**DOI:** 10.3389/fnhum.2021.535512

**Published:** 2021-06-21

**Authors:** Jie Xi, Ge-Tong Wang, Jin Zhao, Chang-Bing Huang

**Affiliations:** ^1^Key Laboratory of Behavioral Science, Institute of Psychology, Chinese Academy of Sciences, Beijing, China; ^2^Department of Psychology, University of Chinese Academy of Sciences, Beijing, China

**Keywords:** binocular disparity, first-order disparity, perceptual learning, second-order disparity, stereopsis, transfer, zero-order disparity

## Abstract

Technological advancements in virtual reality challenge the human vision, especially stereopsis, a function, which characterizes how two eyes coordinate to form a unified three-dimensional (3D) representation of the external world and is found to be deficient in 30% of the normal population. Although a few previous studies have consistently found that the perceptual learning of stereopsis significantly improved stereoacuity, an underlying mechanism of stereo learning remains heavily debated. Here, we trained subjects with normal stereo vision (assessed with the FLY Stereo Acuity Test) to judge stereopsis containing three types of binocular disparity orders (i.e., zero-, first-, and second-order), aiming to systematically examine the characteristics and plasticity of stereo learning. Thirty subjects were randomly assigned to the three training groups (each for the zero-, first-, or second-order disparity separately). The disparity thresholds were measured before and after training. The disparity threshold was measured in 10 additional control subjects only at the pre- and post-training phase. Stereoscopic images were displayed through a shutter goggle, which is synchronized to a monitor. We found that the training significantly improved the zero-, first-, and second-order disparity threshold by 52.42, 36.28, and 14.9% in the zero-order training condition; 30.44, 63.74, and 21.07% in the first-order training condition; and 30.77, 25.19, and 75.12% in the second-order training condition, respectively. There was no significant improvement in the control group. Interestingly, the greatest improvements in the first- and second-order disparity threshold were found in the corresponding disparity training group; on the contrary, the improvements in the zero-order disparity threshold were comparable across all the three disparity training groups. Our findings demonstrated both general (related to the zero-order disparity) and specific improvements (related to the first- and second-order disparity) in stereo learning, suggesting that stereo training occurs at different visual processing stages and its effects might depend on the specific training sites.

## Introduction

**Figure 1:**
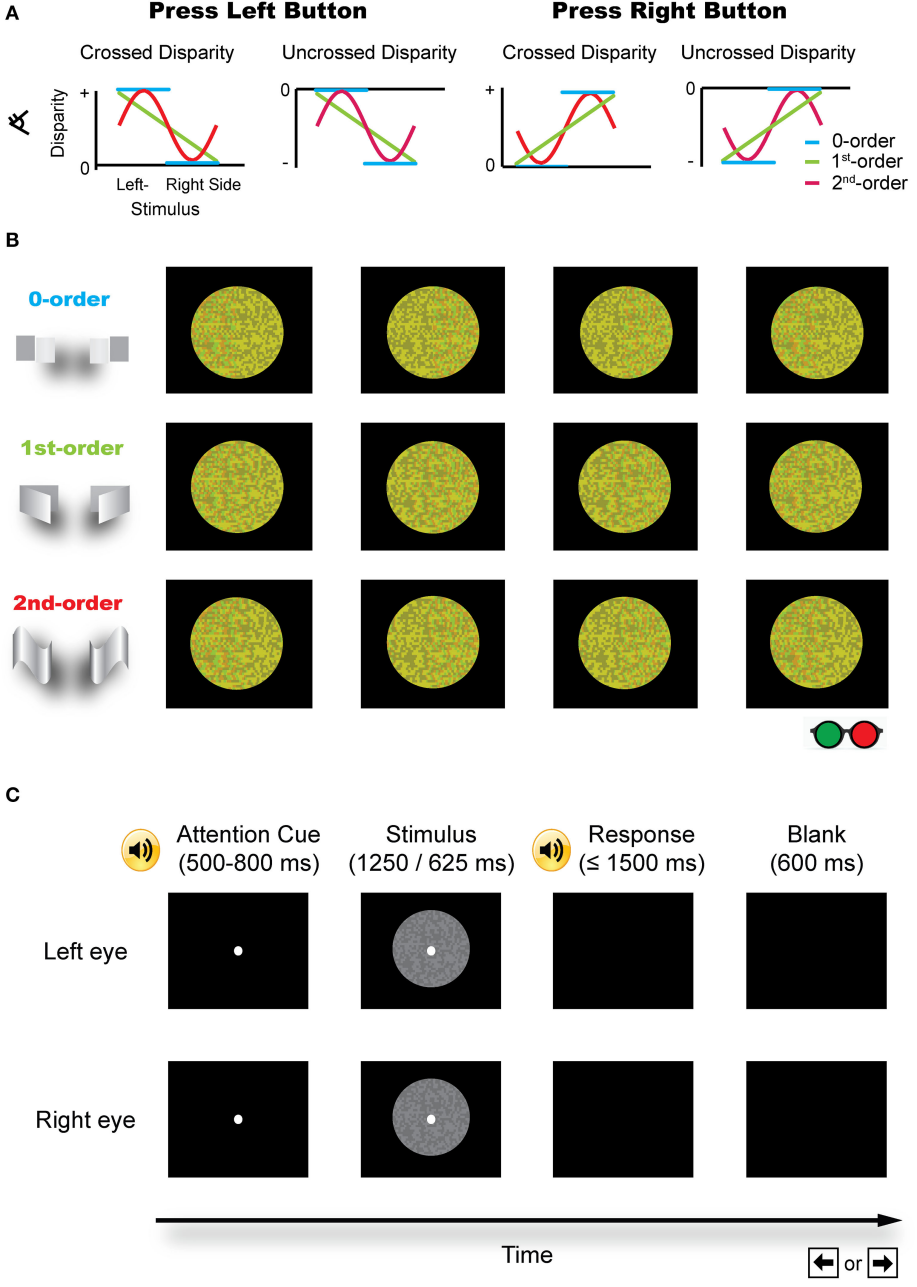
Schematic illustration of the stimuli. **(A)** Different disparity profiles used in the zero-, first-, and second-order disparity condition. Subjects were asked to judge which half of an image was nearer by pressing a left or right button. **(B)** The red-green version of the example stimuli (for illustration purposes). **(C)** A typical trial procedure. Each trial started with an attention cue (500–800 ms). A binocular disparity stimulus was presented for 1,250 or 625 ms. After the response, a blank screen was presented for 600 ms and the next trial started afterward.

Stereopsis is an important process in the perception of our three-dimensional (3D) world. The most important cue to stereopsis (especially for fine stereopsis) is horizontal binocular disparity, which is utilized by visual system in a distributed and hierarchical fashion to retrieve a 3D layout of the external world. Based on how a disparity has been defined (e.g., relative to fixation or an image in the zero plane with zero disparity), it can usually be categorized into either absolute or relative disparity (Cumming and Parker, 1999; Anzai and DeAngelis, 2010; Verhoef et al., 2016). Absolute disparity indicates the distance in depth relative to an observer or a fixation point (near or far) and the relative disparity is defined as the difference in the absolute disparity between the two retinal images. On the other hand, the zero-, first-, or second-order disparity is used to describe the depth structure of a surface (Janssen et al., 2000; Orban, 2011). Specifically, the zero-order disparity is simply a range or distance relative to an observer or a fixation point, and refers to a flat disparity plane. The first-order disparity refers to the depth along an axis in a slanted plane, reflecting a 3D orientation. The second-order disparity describes a 3D shape, which is curved in depth (Figure 1A).

Many regions along a hierarchy of the dorsal and ventral visual pathway exhibit binocular disparity selectivity. Using the functional MRI (fMRI), Neri et al. (2004) found that the processing in the dorsal areas may rely mostly on absolute disparity whereas ventral areas code both relative and absolute disparities. These results are consistent with the view that absolute disparity is useful in providing a rough estimate of the distance of an approaching object to improve visually guided actions (Rashbass and Westheimer, 1961; Erkelens and Collewijn, 1985; Cumming and Judge, 1986) while relative disparity is useful for fine depth judgments about a precise 3D shape in order to facilitate object recognition (Westheimer, 1979; Prince et al., 2000). In addition to a rough dichotomy between the dorsal and ventral visual pathway, the processing of different types of binocular disparity progresses from the lower to higher visual pathway. The early visual cortex mainly signals absolute disparity, suggesting that it may only be involved in the initial stages of binocular disparity computations (Poggio et al., 1985, 1988; Hubel and Livingstone, 1987; Cumming and Parker, 1999). The processing of the second-order disparity information is accomplished at the later stages of the ventral and dorsal visual pathway—that is, the temporal sulcus (TE) (Janssen et al., 2003; Verhoef et al., 2016) and intraparietal sulcus (IPS) (Tsao et al., 2003; Shikata et al., 2008; Georgieva et al., 2009).

The specificity of perceptual learning to the trained stimulus, task, or retinal location in psychophysical studies has been generally taken as evidence for neural plasticity in the early visual cortex (Karni and Sagi, 1991; Gilbert, 1994; Schoups et al., 1995; Watanabe et al., 2002; Chen and Fang, 2011; Crist et al., 2014). Alternatively, recent studies (Liu, 1999; Liu and Weinshall, 2000; Furmanski et al., 2004; Law and Gold, 2008; Xiao et al., 2008; Hua et al., 2010; Zhang et al., 2010) found that learning did transfer to other stimuli and tasks, suggesting that visual perceptual learning (VPL) may be mediated by higher cortical areas. Also, recent models of conscious visual perception suggest the reverse hierarchical processing whereby higher-order areas in the ventral and dorsal stream provide a top-down feedback to the early visual cortex (i.e., predictive coding—Rao and Ballard, 1999; Friston, 2003). In the case of perceptual learning, the reverse hierarchical model suggested the involvement of both early and late stages in VPL and the distribution of changes across the neural system may depend upon the physical stimuli and task (Friston, 2003; Ahissar and Hochstein, 2004; for a review see Hochstein and Ahissar, 2002).

Here, we designed three sets of stimuli, which were random dot stereograms (RDS) matched for a disparity containing different types of disparity order (i.e., the zero-, first-, or second-order disparity), aiming to take advantage of the hierarchical nature of the disparity processing to investigate the specificity/transfer effects in VPL. Although the previous studies consistently found that binocular disparity-based stereopsis was highly plastic in both normal and abnormal vision (Ramachandran, 1976; O'Toole and Kersten, 1992; Sowden et al., 1996; Gantz et al., 2007b; Astle et al., 2011; Ding and Levi, 2011; Xi et al., 2014), the characteristics and the underlying mechanism(s) of stereo training remain debated. Several studies used stimuli with a fixed absolute disparity and found the learning of binocular disparity being specific to retinal location (Ramachandran, 1976; O'Toole and Kersten, 1992), spatial frequency (Long, 1982), or stimulus orientation (Ramachandran and Braddick, 1973). On the other hand, Sowden et al. (1996) found that stereo learning was completely transferred to other retinal locations and proposed that the previous findings of retinal location-specific improvements after disparity training may be due to selective spatial attention. One exception was the finding of a significant improvement at the untrained spatial frequency and orientation after absolute disparity training with Gabor patch (Li et al., 2016). Also, Gantz et al. (2007a) used stimuli with absolute disparity and a rapid learning procedure, i.e., 2,000 trials, and found the training effects being completely transferred to the untrained locations. To our knowledge, these studies have not ruled out a possible confusion regarding the types of disparity (e.g., absolute vs. relative disparity). For example, Sowden et al. (1996) adopted two laterally separated stereograms either above or below the fixation dot and the task was to indicate which of the two stereograms appeared closer. The judgment was based on the relative disparity signal. In addition, all the previous studies of stereo training have used the zero-order disparity. Little is known about the plasticity of the perception of a slanted and curved disparity (i.e., first- and second-order disparity) following intensive training.

In the current study, we trained the subjects to judge stereopsis that was defined by the three types of binocular disparity order (i.e., the zero-, first-, and second-order) and examined the plasticity and transfer effects among the different types of disparity order, aiming to provide a more systematic picture of stereo learning.

## Materials and Methods

### Subjects

Fourty healthy human subjects (22.6 ± 2.7 years; 21 males) participated in the study. All the subjects were right-handed, had no psychiatric or neurological disorders, were naïve to the task, and had a normal or corrected-to-normal visual acuity (monocularly and binocularly, all better than 0.1 logMAR or 6/7.5). Stereoacuity was measured by using the FLY Stereo Acuity Test (Vision Assessment Corporation, Elk Grove Village, IL, USA) before the formal experiment to ensure all the subjects had proper stereopsis (32 arcsec or better). Subjects wore their corrective glasses, if necessary, during the entire experiment. The study was approved by the Ethical Review Committee of the Institute of Psychology, Chinese Academy of Sciences, and an informed consent was obtained from all the subjects. The subjects received subsidies for their participation.

The subjects were randomly and evenly assigned to the three different training groups (training at the zero-, first-, or second-order disparity) and one control group whose disparity threshold was assessed only at the pre- and post-training phase (without training).

### Apparatus

Gamma-corrected stimuli were generated by using a DELL computer running MATLAB (The Mathworks Corp., Natick, MA, USA) and PsychToolbox subroutines (Brainard, 1997; Denis, 1997), and it was presented on an ASUS VG278 3D monitor with a resolution of 1,920 × 1,080 pixels at a refresh rate of 80 Hz. Each pixel subtended 0.46 min of arc at a viewing distance of 2.35 m. Stereoscopic images were displayed dichoptically through liquid crystal shutter glasses (NVIDIA 3D VISION) that were synchronized to the VG 278 3D monitor. The mean luminance of the stimulus and background was 43.86 and 0.26 cd/m^2^, respectively. A chin/forehead rest was used to minimize head movement during the experiment.

### Stimuli and Tasks

The stimuli consist of 1,600 patches (40 × 40), each subtending 4.6 × 4.6 arcmin of visual angle, and being windowed by a circular aperture of 3° diameter. The contrast of the bright and dark dot was 0.4 and 0.6, respectively. The patterns in the two eyes were identical except for a relative horizontal shift (binocular disparity) in the two eyes.

Three sets of RDS were used in the experiments as shown in Figures 1A,B.

(1) The zero-order disparity in which a disparity, crossed (in front of the zero plane) or uncrossed (behind the zero plane), was only added to the left or right half of an image. There were four possible cases: (i) crossed disparity was only added to the left half of the image (the left half appeared in front of the right half and the background); (ii) crossed disparity was only added to the right half of an image (the right half appeared in front of the left half and the background); (iii) uncrossed disparity was only added to the left half of an image (the left half appeared behind the right half and the background); and (iv) uncrossed disparity was only added to the right half of an image (the right half appeared behind the left half and the background).

(2) The first-order disparity was defined by a slant rotated around the left or right edge. Binocular disparity was added based on the specified disparity-defined slant angle and linearly varied over the entire slant of an image. There were also four possible conditions: (i) crossed disparity was added to the left edge of an image, which was extrapolated from left to right, such that the whole pattern appeared in front of the background and the left edge appeared outermost; (ii) crossed disparity was added to the right edge of an image, which was extrapolated from right to left, such that the whole pattern appeared in front of the background and the right edge appeared outermost; (iii) uncrossed disparity was added to the left edge of an image, which was extrapolated from left to right, such that the whole pattern appeared behind the background and the left edge appeared innermost; and (iv) uncrossed disparity was added to the right edge of an image, which was extrapolated from right to left, such that the whole pattern appeared behind the background and the right edge appeared innermost.

(3) The second-order disparity was defined by a vertical sinusoidal cycle or its antiphase counterpart. This condition also consists of four types of stimuli: (i) the crossed disparity varying sinusoidally in depth and the whole pattern appeared in front of the background; (ii) uncrossed disparity varying sinusoidally in depth and the whole pattern appeared behind the background; (iii) crossed disparity varying sinusoidally in antiphase and the whole pattern appeared in front of the background; and (iv) uncrossed disparity varying sinusoidally in antiphase and the whole pattern appeared behind the background.

### Design and Procedures

The experiment consists of a pre-training assessment, stereo training at a particular disparity condition, and a post-training assessment. Before the experiment, we conducted a pilot experiment to determine the suitable exposure duration for each subject. We varied the exposure duration of the stereo stimulus from 125, 62.5, 12.5, 6.25, 1.25, and 0.625 to 0.125 s in descending order and obtained the rough estimates of each disparity threshold within ~50 trials. Because disparities in the fusible fine range are 0.66–13.0 arcmin (Giaschi et al., 2013), we choose the exposure duration that corresponds to a disparity threshold around 13.0 arcmin as the display duration during the threshold assessment and training. The exposure time was 1.25 or 0.625 s for most of the subjects in all the three disparity conditions.

The training consists of eight sessions on separate days, and each session consists of three to six blocks of a hundred trials and lasts for about 30–60 min. Before and after training, the threshold for the three disparity conditions was measured in three separate blocks and counterbalanced across subjects but held constant between the pre- and post-training test sessions for a particular subject. The disparity threshold was measured in 10 additional control subjects only at the pre- and post-training phase.

### Tasks

The same task was used in the pilot experiment, the pre-/post-training stereoacuity measurement, and the stereo training phase (Figure 1C). Each trial started with a 500–800 ms fixation (12 pixels and subtended 5.5 arcmin of visual angle, randomly jittered in time to minimize anticipation) that was signaled by a brief tone, followed by a RDS stimulus. Subjects were instructed to maintain the fixation on a white dot at the center of the display. After the stimulus presentation, the subjects were instructed to indicate which half of the presented stereo stimulus was nearer by pressing the left or right button within 1,500 ms. During the training, a brief tone followed each correct response. During the pilot experiment and the pre-/post-test, a brief tone followed each response regardless of its accuracy. After the response, a blank screen was presented for 600 ms and the next trial started afterward.

A two-down one-up staircase controlled stimulus disparity of each trial, in which two consecutive correct responses resulted in a reduction of disparity [*D*_*n*+1_ = 0.9*D*_*n*_], and one wrong response resulted in an increase in disparity [*D*_*n*+1_ = 1.1*D*_*n*_], converged to a performance level of 70.7% correctness. All the disparities were expressed in log units of pixels (0.46 arcmin/pixel) and were rounded to their closest integer values. A reversal was obtained as a result when the staircase changed its direction (changing from an increasing to a decreasing disparity or vice versa). According to the standard psychophysical practice, the first three (if there were an odd number of total reversals) or four (if even) reflections were discarded and the average of the remaining reversals was taken as the threshold.

In both pre- and post-training assessments, visual acuity and stereoacuity for the zero-, first-, or second-order disparity were measured for all the observers, taking up to a total of ~1 h. Visual acuity was measured monocularly (left/right eye) and binocularly by using the Chinese Tumbling E Chart (Mou, 1966; Xi et al., 2014) and defined as the logMAR acuity associated with the 75% correct identification.

### Statistical Analysis

Data were tested for normal distribution by using the Kolmogorov–Smirnov test. The pre- and post-training disparity threshold was compared by using a one-way repeated ANOVA (pre- vs. post-training) with the Greenhouse–Geisser correction. The value of *p* < 0.05, between 0.05 and 0.10, and > 0.10 was defined as significance, marginal significance, and non-significance, respectively.

For each observer, the percentage of improvement for all the three measures (disparity threshold and visual acuity) was calculated as

$$I=\frac{\text{Measur}{\text{e}}_{\text{pre}\text{-}\text{training}}-\text{Measur}{\text{e}}_{\text{post}\text{-}\text{training}}}{\text{Measur}{\text{e}}_{\text{pre}\text{-}\text{training}}}\;\times \;100\%$$

The learning rate [i.e., disparity threshold as a function of the training session (in log units) for each observer and the group average] was fitted with a linear function:

$$\text{Log}(D)=\text{log}(D_{0})+\alpha \text{log }\left(\text{Session}\right)$$

where *D* denotes the disparity threshold and α is the slope of the learning rates.

## Results

**Figure 2:**
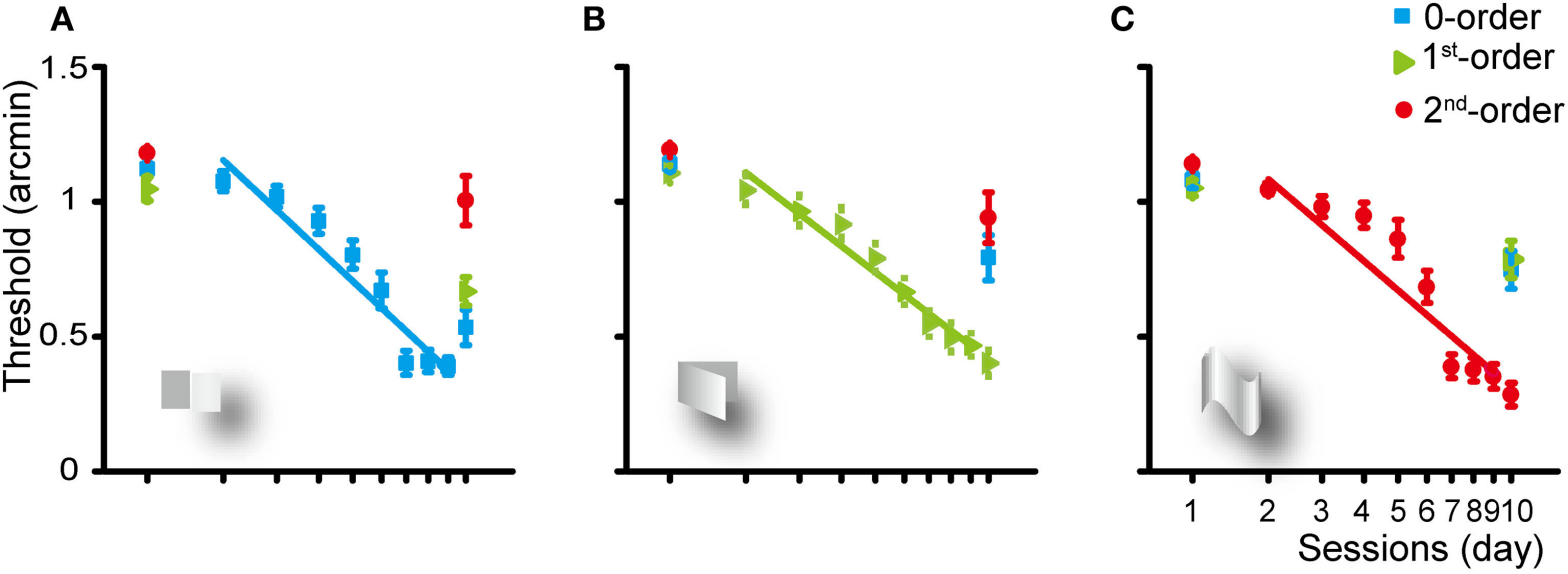
Group average learning rate for the zero- **(A)**, first- **(B)**, and second-order **(C)** disparity training condition. The learning rate was fitted with a linear function. Error bars represent standard error across subjects. The first and last data points were derived from the pre- and post-training assessments.

In general, the disparity threshold decreased significantly in all the three training groups (Figure 2) and the improvement transferred to untrained disparity conditions. There was no significant improvement in visual acuity in the left/right eye or binocular vision after training (all *p* > 0.1). The Kolmogorov–Smirnov normal distribution test showed that the pre- and post-test threshold of the three types of disparity order is normally distributed (all *p* > 0.05).

### Zero-Order Disparity Training

Training of the subjects for 8 days for the detection of the zero-order disparity significantly improved the zero-order disparity threshold at a rate of 1.16 log arcmin per log unit of training session (*p* < 0.001). Averaged across the subjects, the zero-order disparity threshold improved from 1.12 to 0.53 log arcmin [a mean reduction of 52.42%, SE: 11.29%; *F*_(1, 9)_ = 51.834, *p* < 0.001, η_*p*_^2^ = 0.852]. In addition, the first-order disparity threshold decreased significantly from 1.05 to 0.67 [a reduction of 36.28%, SE: 14.03%; *F*_(1, 9)_ = 18.173, *p* < 0.01, η_*p*_^2^ = 0.675] and the second-order disparity marginally significantly decreased from 1.18 to 1 [a reduction of 14.9%, SE: 15.44%; *F*_(1, 9)_ = 4.855, *p* = 0.055, η_*p*_^2^ = 0.350] log arcmin, manifesting a generalization from the zero-order disparity training to other forms of disparity processing.

### First-Order Disparity Training

The first-order disparity training significantly improved the first-order disparity threshold at a rate of 1.15 log arcmin per log training session (*p* < 0.001). Averaged across the subjects, the disparity threshold decreased significantly from 1.14 to 0.79 [a reduction of 30.44%, SE: 15.03%; *F*_(1, 9)_ = 13.604, *p* < 0.01, η_*p*_^2^ = 0.602], 1.11 to 0.4 [a reduction of 63.74%, SE: 9.13%; *F*_(1, 9)_ = 88.097, *p* < 0.001, η_*p*_^2^ = 0.907], and 1.19 to 0.94 [a reduction of 21.07%, SE: 15.8%; *F*_(1, 9)_ = 7.997, *p* < 0.05, η_*p*_^2^ = 0.471] log arcmin for the zero-, first-, or second-order disparity after the first-order disparity training, respectively.

### Second-Order Disparity Training

The second-order disparity training significantly improved the second-order disparity threshold at a rate of 1.26 log arcmin per log training session (*p* < 0.001). Averaged across the subjects, the disparity threshold decreased significantly from 1.08 to 0.75 [a reduction of 30.77%, SE: 13.87%; *F*_(1, 9)_ = 15.824, *p* < 0.01, η_*p*_^2^ = 0.637], 1.05 to 0.79 [a reduction of 25.19%, SE: 13.48%; *F*_(1, 9)_ = 12.637, *p* < 0.01, η_*p*_^2^ = 0.584], and 1.14 to 0.28 [a reduction of 75.12%, SE: 7.07%; *F*_(1, 9)_ = 153.275, *p* < 0.001, η_*p*_^2^ = 0.945] log arcmin for the zero-, first-, or second-order disparity after the second-order disparity training, respectively.

### Comparison Between Crossed and Uncrossed Disparity

We compared the crossed- and uncrossed-disparity threshold from the pre- and post-training test session and the slope of the averaged best fitting rates of the three types of disparity order. For the first-order disparity training group, the crossed-disparity threshold was significantly larger than the uncrossed-disparity threshold in the pre-training test sessions [*F*_(1, 9)_ = 6.291, *p* = 0.033, η_*p*_^2^ = 0.411]. There was no other difference between the crossed- and uncrossed-disparity threshold or slope of the averaged best fitting rates (all *p* > 0.1).

### Comparison Between the Different Disparity Training Group

**Figure 3:**
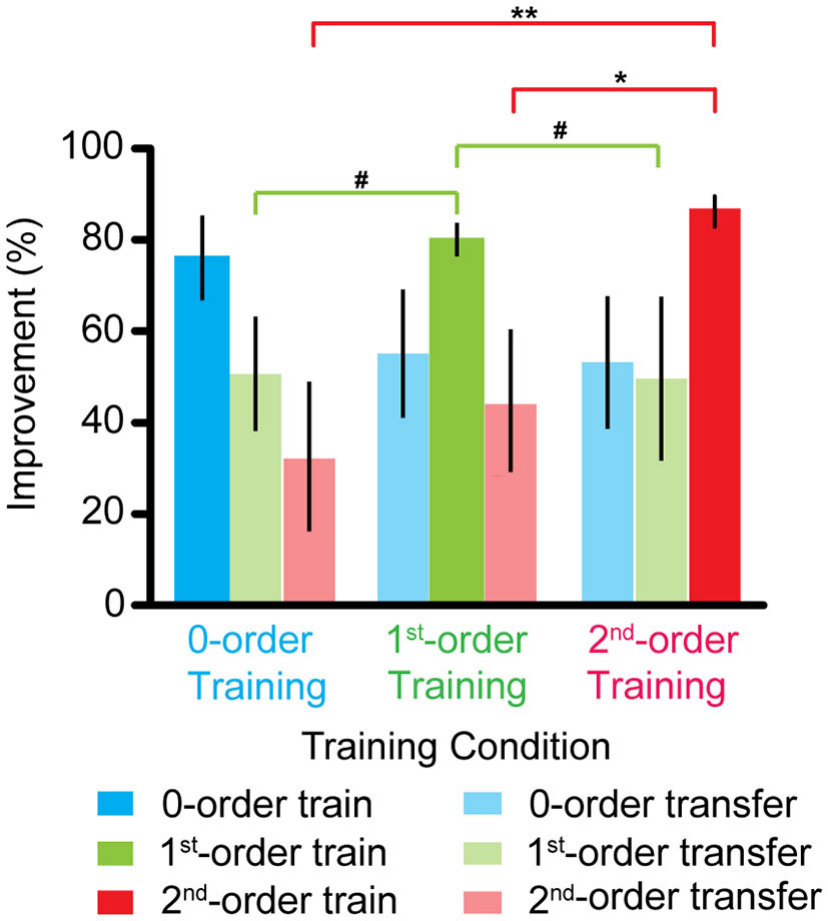
Improvements of the zero-, first-, and second-order disparity threshold after the zero-, first-, and second-order disparity training. Error bars represent standard error across the subjects. ^*^p < 0.05; ^**^p < 0.01; ^#^0.05 < *p* < 0.10.

Figure 3 shows that an improvement in the zero-order disparity threshold was comparable across all the three training groups (all *p* > 0.1). However, the first-order disparity threshold improvement in the first-order disparity training group was marginally greater than that in the zero- and second-order disparity training group [first- vs. zero-order training: *F*_(1, 9)_ = 4.470, *p* = 0.064, η_*p*_^2^ = 0.332; first- vs. second-order training: *F*_(1, 9)_ = 3.455, *p* = 0.096, η_*p*_^2^ = 0.278]. Furthermore, the second-order disparity threshold improvement in the second-order disparity training group was significantly greater than that in the zero- and first-order disparity training group [second- vs. zero-order training: *F*_(1, 9)_ = 12.025, *p* < 0.01, η_*p*_^2^ = 0.572; second- vs. first-order training: *F*_(1, 9)_ = 9.425, *p* < 0.05, η_*p*_^2^ = 0.512]. Our results indicate separate mechanisms and plasticity in the processing of the first- and second-order disparity information, and the zero-order disparity processing seems to precede the first- and second-order disparity processing.

We also compared the training effects for the three types of disparity order within each training group. For the zero-order disparity training group, an improvement in the zero-order disparity threshold was significantly greater than in the first- and second-order disparity [zero- vs. first-order disparity: *F*_(1, 9)_ = 6.252, *p* < 0.05, η_*p*_^2^ = 0.410; zero- vs. second-order training: *F*_(1, 9)_ = 5.712, *p* < 0.05, η_*p*_^2^ = 0.388]. There was no difference between the first- and second-order disparity [first- vs. second-order disparity: *F*_(1, 9)_ = 1.508, *p* > 0.5]. For the first-order disparity training group, the first-order disparity threshold improvement was significantly greater than that in the zero-order disparity [*F*_(1, 9)_ = 8.650, *p* < 0.05, η_*p*_^2^ = 0.490] and marginally significant than that in the second-order training [*F*_(1, 9)_ = 4.597, *p* = 0.061, η_*p*_^2^ = 0.338]. There was no difference between the zero- and second-order disparity [*F*_(1, 9)_ = 0.560, *p* > 0.5]. For the second-order disparity training group, the second-order disparity threshold improvement was significantly greater than that in the zero- [*F*_(1, 9)_ = 12.280, *p* < 0.01, η_*p*_^2^ = 0.577] and first-order training [*F*_(1, 9)_ = 13.691, *p* < 0.01, η_*p*_^2^ = 0.603]. Again, there was no difference between the zero- and first-order disparity improvement [*F*_(1, 9)_ = 0.003, *p* > 0.5].

### Control Experiment

In the control group, there was no significant improvement in any measurements. The disparity threshold in the pre-test was 1.16, 1.10, and 1.17 log arcmin, which was changed to 1.12, 1.00, and 1.15 log arcmin in the post-test for the zero-, first-, and second-order disparity (all *p* > 0.5), respectively.

## Discussion

In the present study, we evaluated the effects of perceptual learning with the three types of binocular disparity order. Three groups of subjects participated in either the zero-, first-, or second-order disparity training. The disparity threshold of all three types of binocular disparity order was measured before and after the training. Our results show that the disparity threshold decreased significantly in all the three training groups (i.e., the zero-, first-, or second-order) in the trained condition and was transferred to other control conditions. Interestingly, the greatest improvements in the first- and second-order disparity threshold were found in the corresponding disparity training group; on the contrary, the improvements in the zero-order disparity threshold were comparable across all the three disparity training groups. Finally, no significant improvement was observed in the control subjects.

Substantial research has sought to specify the underlying neural and functional mechanisms for perceptual learning. Some argue that VPL is mediated by higher cortical areas and suggested the involvement of both early and later stages in VPL (Liu, 1999; Liu and Weinshall, 2000; Furmanski et al., 2004; Law and Gold, 2008; Xiao et al., 2008; Hua et al., 2010; Zhang et al., 2010). On the other hand, the specificity of perceptual learning to the trained stimulus, task, or retinal location in VPL (Karni and Sagi, 1991; Gilbert, 1994; Schoups et al., 1995; Watanabe et al., 2002; Crist et al., 2014) was taken as evidence for neural plasticity in the early visual cortex (Schwartz et al., 2002; Furmanski et al., 2004; Jehee et al., 2012). Interestingly, in the current study, we found that the zero-order disparity threshold decreased significantly and comparably among the three different training groups, while the first- and second-order disparity threshold improved most prominently in the corresponding training group, demonstrating both general (related to the zero-order disparity) and specific (related to the first- and second-order disparity) learning effects in the perceptual learning of stereo judgment. These results may have reconciled some of the inconsistencies in the literature (Ramachandran and Braddick, 1973; Ramachandran, 1976; Long, 1982; O'Toole and Kersten, 1992; Sowden et al., 1996). While several studies used stimuli with a fixed absolute disparity and found specific binocular disparity training effects, Sowden et al. (1996) used relative disparity and found that the training effect was completely transferred to other retinal locations. The discrepancy between these results might be due to the different types of disparity order they used. The zero-order training may occur at an early site and is highly specific to the retinotopic location and characteristics of the trained stimulus. The first- and second-order training may depend on the higher sites as well as the earlier sites that process the zero-order disparity, resulting in a more generable learning process(es). To our knowledge, this is the first study to systematically examine the effects of training on the different types of binocular disparity order.

The distributed hierarchical processing of stereo disparity has made training on different types of binocular disparity orders, which may be particularly informative in investigating the specificity and transfer effects of visual perceptual learning. The observed specific learning effects may indicate that the lower-order disparity is likely to provide antecedent representations for the higher-order disparity processing. While not directly testing this hypothesis in the current study, these specific learning effects were consistent with the human fMRI studies, which found that the processing of different types of binocular disparity order also progresses from lower to higher visual pathways (Poggio et al., 1985, 1988; Hubel and Livingstone, 1987; Cumming and Parker, 1999; Janssen et al., 2003; Tsao et al., 2003; Shikata et al., 2008; Georgieva et al., 2009; Verhoef et al., 2016). The early visual cortex mainly signals the absolute and zero-order disparity and may only be involved in an initial stage of binocular disparity computations (Poggio et al., 1985, 1988; Hubel and Livingstone, 1987; Cumming and Parker, 1999). The processing of the second-order disparity information is accomplished at the later stages of the ventral and dorsal visual pathways—that is, the TE (Janssen et al., 2003; Verhoef et al., 2016) and IPS (Tsao et al., 2003; Shikata et al., 2008; Georgieva et al., 2009).

Various models suggest that the specificity in perceptual learning can arise from training-induced modifications of the recurrent horizontal connections in V1, leading to sharpened neuronal tuning (Adini et al., 2002; Teich and Qian, 2003; Zhaoping et al., 2003), from an improved readout (of V1 signals) through the response reweighting within the visual cortex (in decision areas) (Poggio et al., 1992; Dosher and Lu, 1998) or through top-down processes (Li et al., 2008). The observed specificity in our study is in line with the reverse hierarchical model that proposed that the degree of specificity depends on the difficulty of training conditions (Ahissar and Hochstein, 1997, 2004; Hochstein and Ahissar, 2002), and suggested that different types of stereo training may occur at different training sites: the zero-order training may occur at one site while the first- and second-order training may occur at multiple sites, including a site, which processes the zero-order disparity.

The subject's task was to indicate which half of the presented stimulus was nearer by pressing a left or right arrow button. To perform this task, the subjects must perceive the whole plane of the depth profiles. However, in the zero-order disparity condition, the crossed or uncrossed disparity was only added either to the left or to the right half of an image, and the other half was maintained at zero disparity. The unmatched disparity magnitudes between the left and right parts of the zero-order stimuli might induce vergence eye movements, hence providing a non-disparity cue to solve the task. We derived the crossed- and uncrossed-zero-order disparity threshold and slope of the averaged best fitting curves by applying a maximum-likelihood method to the pre- and post-training test data. There was no significant difference between the crossed- and uncrossed-zero-order disparity threshold in the pre- and post-training session or slope of the averaged best fitting curves, indicating the modest contribution, if any, of non-disparity cues in our tasks.

## Conclusion

Our findings suggest both general (related to the zero-order disparity) and specific improvements (related to first- and second-order disparity) in stereo learning, suggesting that stereo training may occur at different visual processing stages and that its effects might depend on the specific training sites. This is the first report on the stereopsis training that used the three types of binocular disparity order (i.e., the zero-, first-, and second-order). Further studies should attempt to explore the cortical activation and organization underlying this general and specific effect after the disparity training. For practical purposes, it would also be interesting for future studies to use a multistage training protocol to design the most efficient training strategy (Li et al., 2016).

## Data Availability Statement

The raw data supporting the conclusions of this article will be made available by the authors, without undue reservation, to any qualified researcher.

## Ethics Statement

The studies involving human participants were reviewed and approved by Ethical Review Committee of Institute of Psychology, Chinese Academy of Sciences. The patients/participants provided their written informed consent to participate in this study.

## Author Contributions

JX and C-BH designed the experiment and wrote the manuscript. JX, JZ, and G-TW collected the data. JX and G-TW conducted the analyses. G-TW edited the manuscript. All authors contributed to the article and approved the submitted version.

## Conflict of Interest

The authors declare that the research was conducted in the absence of any commercial or financial relationships that could be construed as a potential conflict of interest.
